# The impact of PSMA-PET/CT on clinical decision-making in primary staging of prostate cancer

**DOI:** 10.1186/s13550-026-01480-2

**Published:** 2026-06-30

**Authors:** Jacob Ingvar, Rasmus Green, Elin Trägårdh, Anders Bjartell

**Affiliations:** 1 Department of Urology, Skåne University Hospital, Jan Waldenströmsgata 5, Malmö, 205 02 Sweden; 2 Department of Translational Medicine, Lund University, Malmö, Sweden; 3 Clinical Physiology and Nuclear Medicine, Skåne University Hospital, Malmö, Sweden; 4 Wallenberg Centre for Molecular Medicine, Lund University, Lund, Sweden

**Keywords:** PSMA-PET/CT, Prostate cancer, Primary staging, Clinical decision-making, Treatment management

## Abstract

**Background:**

Accurate staging is essential for optimal management of prostate cancer. Conventional imaging with computed tomography and bone scintigraphy has limited sensitivity and specificity, particularly for small-volume disease. Prostate-specific membrane antigen positron emission tomography/computed tomography (PSMA-PET/CT) has emerged as a more sensitive modality, but its impact on clinical decision-making in primary staging remains to be fully defined. In this retrospective single-center study, we evaluated the impact of PSMA-PET/CT on treatment strategies compared with conventional imaging in patients with intermediate- and high-risk prostate cancer at Skåne University Hospital, Sweden, between October 2020 and December 2022. All patients underwent [^18^F]PSMA-1007 PET/CT, computed tomography and bone scintigraphy within 90 days. Management decisions were assessed by comparing treatment decisions based on conventional imaging alone with those incorporating PSMA-PET/CT findings, and were classified as major (shift between curative and non-curative treatment intent), medium (modification of surgical or radiotherapy plans without altering overall treatment intent), and minor (additional investigations or minor adjustments resulting in treatment delay without change of intent or modality).

**Results:**

A total of 287 consecutive patients were included, of whom 59 (21%) had intermediate-risk and 228 (79%) high-risk disease. Overall, 187 patients (65%) had negative findings on all imaging modalities, while 100 (35%) had positive findings. PSMA-PET/CT identified lesions in 94 patients, whereas only 6 patients had positive findings exclusively on conventional imaging. PSMA-PET/CT led to a change in clinical management in 65 patients (22.6%), including major changes in 31 (10.8%), medium changes in 28 (9.9%), and minor changes in 6 (2.1%). Among patients with positive PSMA-PET/CT findings (*n* = 94), 32.0% experienced major, 29.8% medium and 5.2% minor treatment modifications. Management changes were more frequent in high-risk patients (24.6%) compared to intermediate-risk patients (15.3%).

**Conclusion:**

PSMA-PET/CT provides actionable information that leads to changes in clinical management in approximately one in four patients undergoing primary staging of prostate cancer, with the greatest impact observed in high-risk disease. These findings support the integration of PSMA-PET/CT into routine staging to enable more precise and individualized treatment planning.

## Introduction

Prostate cancer is one of the most common malignancies worldwide, and accurate staging is a cornerstone of appropriate treatment selection and prognosis [1, 2]. Current guidelines are not uniform regarding imaging for staging of prostate cancer, with some recommending conventional imaging using computed tomography (CT) and bone scintigraphy (BS), while others support the use of PSMA-PET/CT in men with intermediate- and high-risk prostate cancer [3, 4]. BS is highly sensitive for advanced large osteoblastic lesions but has limited specificity, with uptake in degenerative or traumatic bone changes potentially leading to false positives and also deficient sensitivity in small metastatic lesions and early marrow disease not being detected [5, 6]. Similarly, CT has poor sensitivity for detecting lymph node metastases, especially those smaller than 8–10 mm, resulting in a significant risk of under-staging [7].

Among the most promising new innovations in prostate cancer diagnostics is prostate-specific membrane antigen positron emission tomography combined with computed tomography (PSMA-PET/CT). A growing body of evidence has confirmed that PSMA-PET/CT outperforms CT and BS in sensitivity and specificity for both nodal and bone metastases [8–10].

Beyond its diagnostic superiority, the key clinical question is whether PSMA-PET/CT leads to any changes in patient management. Several prospective and randomized studies suggest that the enhanced detection capability of PSMA-PET/CT translates to a different therapeutic strategy. However, most of them are in the biochemical recurrence (BCR)-setting, like the CONDOR trial which showed a 64% management change due to PSMA-PET/CT [11].

In the proPSMA trial, a prospective and randomized staging study, PSMA-PET/CT demonstrated a 27% greater accuracy than conventional imaging, resulting in a change in management in more than one-quarter of patients with high-risk prostate cancer [9]. Similarly, a prospective study with 73 patients showed that staging with PSMA-PET/CT led to major management change in 16.5% and treatment modifications in 37% of patients planned for curative radiation, respectively [12]. More recently, the phase 2/3 THUNDER trial demonstrated that PSMA-PET/CT leads to significant stage migration compared with conventional imaging in high-risk patients referred for radiotherapy, resulting in clinically relevant changes in treatment planning [13]. In a study from 2024, the inclusion of PSMA-PET/CT in the diagnostic algorithm for patients with intermediate-risk prostate cancer impacted patient management in 13.3% of the cases [14].

This potential to refine therapeutic decision-making underscores the clinical relevance of implementing PSMA-PET/CT in routine practice. A few years ago, key opinion leaders recommended against the use of PSMA-PET/CT mostly due to lacking scientific evidence of long term benefits [15], but more recently, experts voted for a widespread use of PSMA-PET/CT at the APCCC consensus meeting in 2024 [16] and at the European Association of Nuclear Medicine (EANM) consensus meeting in 2023 [17].

The purpose of this study is to explore whether the adoption of PSMA-PET/CT has meaningfully changed treatment decisions compared to the longstanding standard of BS and CT, in the staging of prostate cancer in patients who underwent all three modalities as part of their diagnostic work-up.

## Material & methods

### Study design and patient selection

We retrospectively evaluated a consecutive series of patients who underwent PSMA-PET/CT imaging for primary staging of intermediate or high-risk prostate cancer, from October 2020 to December 2022 at Skåne University Hospital in Southern Sweden. All patients also had a BS within 90 days of the PSMA-PET/CT and most also underwent a contrast-enhanced CT of the chest and abdomen within the same time frame. In 12 patients (4.2%), a low-dose CT acquired as part of the PET/CT examination was used for anatomical assessment because no contrast-enhanced CT had been performed. Lymph nodes > 8 mm in maximum short axis diameter were regarded as positive in accordance with conventional radiological criteria.

A retrospective analysis of the included patients’ medical records was performed by an experienced urologist (JI), including information from multidisciplinary meetings. Patient data, including age, date of diagnosis, treatment modality, PSA level, Gleason score, and ISUP grade, were collected. The findings from PSMA-PET/CT, BS and CT were categorized as either positive or negative for nodal and bone metastases. In case of incomplete or ambiguous information, an experienced radiologist/nuclear physician (ET) re-evaluated the images. For patients with positive findings, a hypothetical management strategy based solely on conventional imaging (CT and bone scintigraphy) was retrospectively reconstructed and subsequently compared with the actual management decision incorporating PSMA PET/CT findings. Intermediate-risk disease was defined as PSA 10–20 ng/mL and/or ISUP grade group 2–3 and/or cT2b-c disease, while high-risk disease was defined as PSA > 20 ng/mL and/or ISUP grade group 4–5 and/or ≥cT3 disease according to the EAU classification [18].

The study protocol was approved by the Regional Ethics Committee in Lund (2016/417, 2018/117, 2018/753, 2020–02524), and the study was conducted in accordance with the Declaration of Helsinki. Informed consent was obtained from all participants prior to study inclusion.

### Imaging

CT was performed as either contrast-enhanced CT of diagnostic quality (100 kV/80–480 mA; noise index 37.5; slice thickness 0.625 mm; iterative reconstruction) or as low-dose CT (120 kV/30–160 mA; noise index 29; slice thickness 3.75 mm; iterative reconstruction). BS was performed by injecting 570 MBq of 99mTc-hydroxydiphosphonate followed by an accumulation time of 2–4 h. Patients were scanned on either Siemens Symbia, a GE Discovery NMCT 670 or a GE Discovery NMCT 870 CZT and planar whole-body anterior and posterior images were acquired. A SPECT/CT could be obtained according to clinical routine, when indicated. Patients were injected with 4 MBq/kg [^18^F]PSMA-1007 and underwent a head-knee PET/CT scan from the base of the skull to the mid-thighs on a GE Discovery MI PET-CT system (Discovery MI; GE Healthcare, Milwaukee, WI, USA) 120 min post-injection. Data were reconstructed using the Q.Clear (GE Healthcare, Milwaukee, WI, USA) algorithm with a β-value of 800, including time-of-flight, point spread function and CT-based attenuation correction with a 256 × 256 matrix (pixel size 2.7 × 2.7 mm^2^, slice thickness 2.8 mm).

Definition of management changes.

Management changes were defined as differences between decisions based on PSMA-PET/CT findings and those based on CT and BS alone. Management changes were classified as major when the treatment intent shifted from curative to non-curative, medium when adjustments involved modification of surgical or radiotherapy plans without altering overall treatment intent, and minor when additional imaging investigations, delayed surgery or radiotherapy due to clarification of equivocal findings, and MDT re-discussion without alteration of overall treatment intent or treatment modality.

## Results

### Patient characteristics

A total of 287 prostate cancer patients were included in the analysis. The mean age was 72 years (range 46–85). Fifty-nine patients (21%) were classified as *intermediate-risk* and 228 (79%) as *high-risk* prostate cancer. The median PSA at diagnosis for the entire cohort was 23.7 ng/mL (range 1.4–517). Clinical characteristics are detailed in Table 1.

**Table 1:** Patient characteristics

Characteristics	All patients (*n* = 287)	Intermediate-risk (*n* = 59)	High-risk (*n* = 228)
**Age (years)** Mean (range)	72.2 (46–85)	70.0 (47–85)	72.4 (46–85)
**PSA at diagnosis (ng/mL)** Median (range)	23.7 (1.4–517)	9.4 (1.7–18)	27.5 (1.4–517)
**ISUP grade group**,** n (%)**			
Grade 1	1 (0.3)	0 (0)	1 (0.4)
Grade 2	34 (11.8)	22 (37.3)	12 (5.3)
Grade 3	51 (17.8)	37 (62.7)	14 (6.1)
Grade 4	51 (17.8)		51 (22.4)
Grade 5	150 (52.3)		150 (65.8)

### Imaging findings

Overall, 187 patients (65%) had negative findings across all imaging modalities, while 100 patients (35%) had positive findings on at least one modality. Among patients with positive imaging findings (*n* = 100), 94% were detected by PSMA-PET/CT, whereas only 6% were identified exclusively by conventional imaging (Table 2). Five of the six cases consisted of small bone lesions identified on bone scintigraphy that were considered suspicious for metastatic disease on conventional imaging but showed no corresponding PSMA-avid lesion on PET/CT. Following multidisciplinary review, these findings were considered non-metastatic. The remaining case involved a small miscellaneous lesion on CT without corresponding PSMA-avid lesion on PET/CT.

**Table 2:** Imaging findings

Imaging findings	All patients *n* (%)	Intermediate-risk *n* (%)	High-risk *n* (%)
Negative PSMA-PET/CT and negative BS/CT	187 (65.2)	46 (78.0)	141 (61.8)
Negative PSMA-PET/CT but positive BS/CT	6 (2.1)	0 (0)	6 (2.6)
Positive PSMA-PET/CT and positive BS/CT	44 (15.3)	3 (5.1)	41 (18.0)
Positive PSMA-PET/CT but negative BS/CT	50 (17.4)	10 (16.9)	40 (17.5)

### Management changes–overall cohort

**Fig. 1:**
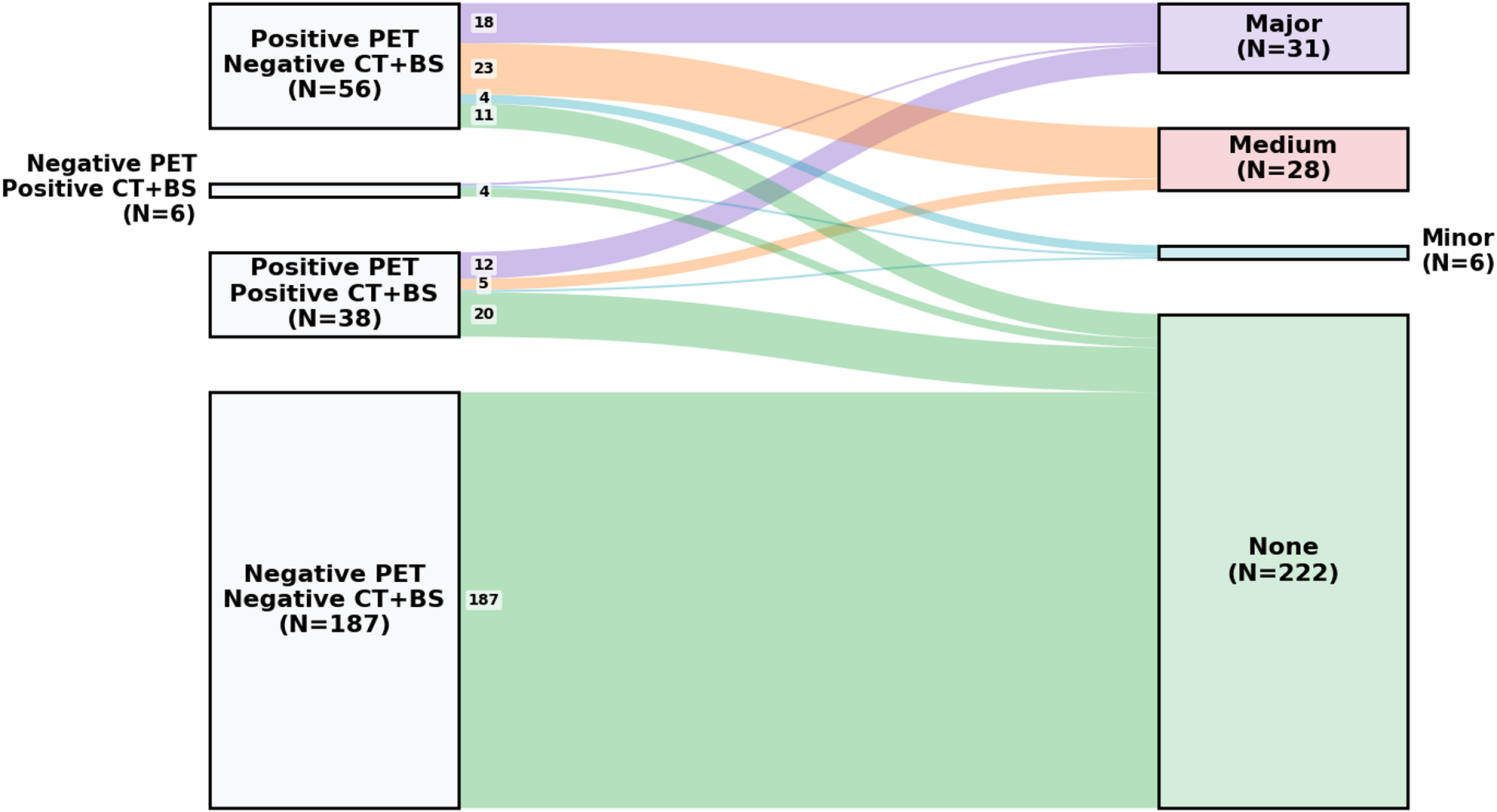
Impact of management changes following PSMA-PET/CT. The diagram illustrates the distribution of patients from imaging findings to management outcomes

PSMA-PET/CT led to a change in clinical management in 65 of 287 patients (22.6%). Major changes occurred in 31 patients (10.8%), medium changes in 28 patients (9.8%), and minor changes in 6 patients (2.1%). Management changes based on PSMA-PET/CT findings are summarized in Table 3, with an overview illustrated in Fig. 1. Table 4 shows examples of management changes for selected patients.

**Table 3:** Impact on clinical management

Management outcome	All patients *n* (%)	Intermediate-risk *n* (%)	High-risk *n* (%)
No change in management	222 (77.4)	50 (84.7)	172 (75.4)
Minor change	6 (2.1)	2 (3.4)	4 (1.8)
Medium change	28 (9.8)	3 (5.1)	25 (11.0)
Major change	31 (10.8)	4 (5.8)	27 (11.8)
Any management change	65 (22.6)	9 (15.3)	56 (24.6)

**Table 4:** Examples of management changes

Patient	Initial treatment	Post PET/CT treatment	Management change	Reason for management change
1	Prostatectomy	ADT+ARPI	Major	Several bone metastases on PET/CT
2	Curative radiation	ADT+ARPI+palliative radiation	Major	7 lymph node metastases in the abdomen on PET/CT
3	Curative Radiation	Curative Radiation with radiation of iliac lymph nodes	Medium	2 iliac lymph node metastases on PET/CT
4	Prostatectomy	Prostatectomy	Minor	Delayed operation due to investigation of indeterminate bone uptake on PET/CT

Among the major changes, treatment intent shifted from curative to non-curative intent in 29 patients and from non-curative to curative in 2 patients. In the 29 patients who were upstaged, PSMA-PET/CT identified metastatic disease not detected on conventional imaging, leading to a change from planned curative treatment to systemic therapy with non-curative intent. Conversely, in the two patients whose treatment intent changed from non-curative to curative, suspicious lesions identified on conventional imaging were not confirmed on PSMA-PET/CT. Following multidisciplinary review, these findings were considered benign or non-metastatic, allowing curative treatment to proceed. In addition, 35 patients (12.2%) had imaging findings on PSMA-PET/CT that did not result in any change in management compared with decisions based on conventional imaging. In most of these cases, metastatic disease had already been identified on conventional imaging, and the PSMA-PET/CT findings therefore did not alter the planned treatment strategy.

**Fig. 2:**
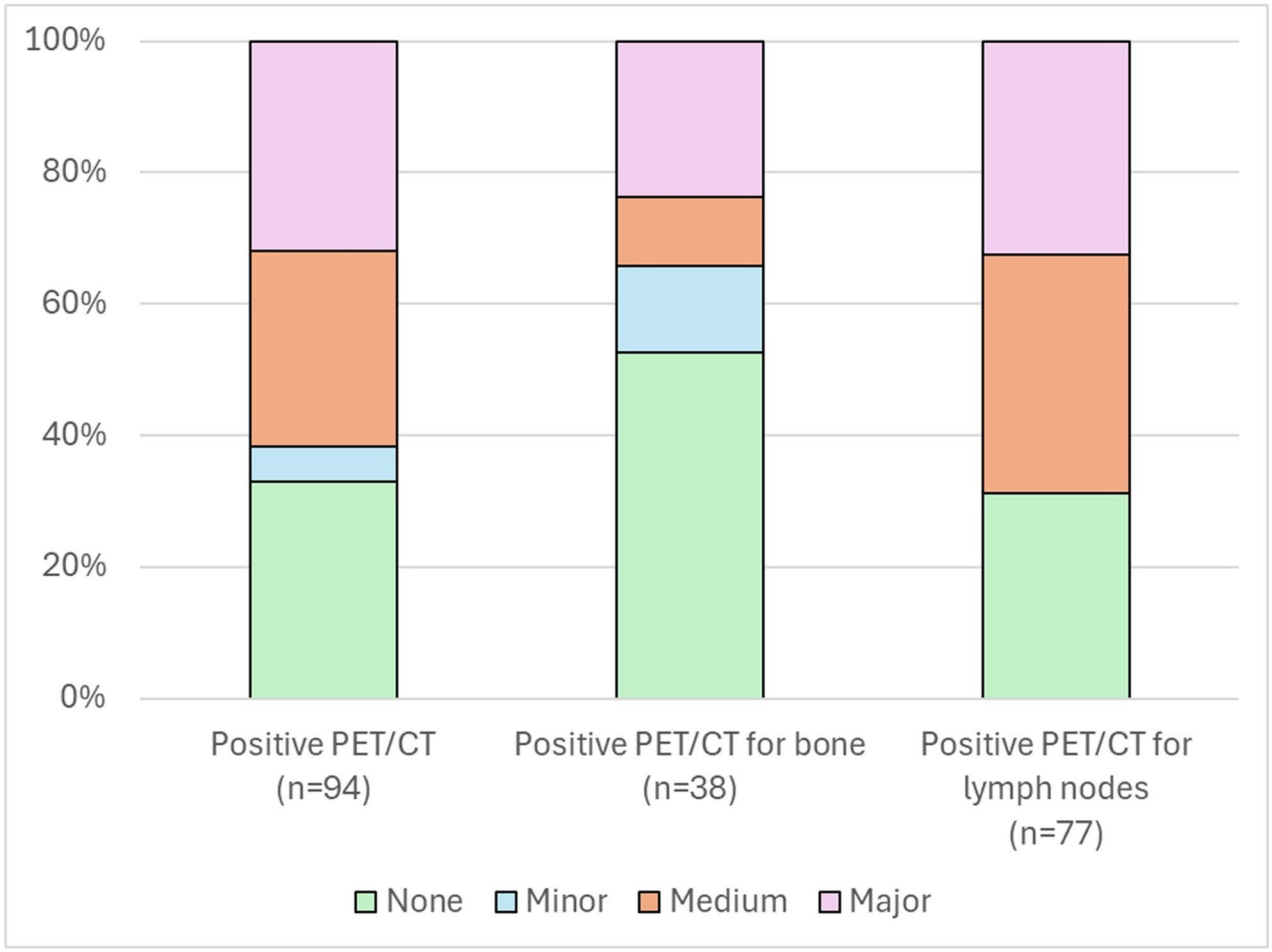
Management changes following PSMA-PET/CT stratified by location of positive PET/CT. Stacked bar chart showing the proportion of patients with no change, minor, medium, and major management changes in patients with positive PET/CT as well as divided into those positive for bone metastases and lymph node metastases

Among the 94 patients with positive PSMA-PET/CT findings, management changes were observed in a substantial proportion. Major changes occurred in 32.0% of patients, medium changes in 29.8%, and minor changes in 5.2%. Figure 2 illustrates the distribution of management changes among patients with positive findings overall on PSMA-PET/CT, as well as stratified by presence of lymph node and bone metastases.

### Subgroup analysis

In the intermediate-risk subgroup (*n* = 59), the median PSA was 9.4 ng/mL (range 1.7–18). Forty-six patients (78%) had negative findings across all imaging modalities, while 13 patients (22%) had positive findings. Management changes were observed in a minority of patients: minor changes in 2 patients (3.4%), medium changes in 3 patients (5.1%), and major changes in 4 patients (6.8%). In addition, 4 patients (6.8%) had positive findings that did not lead to any modification in clinical management, for example non-curative findings in both PSMA-PET/CT and conventional imaging.

**Fig. 3:**
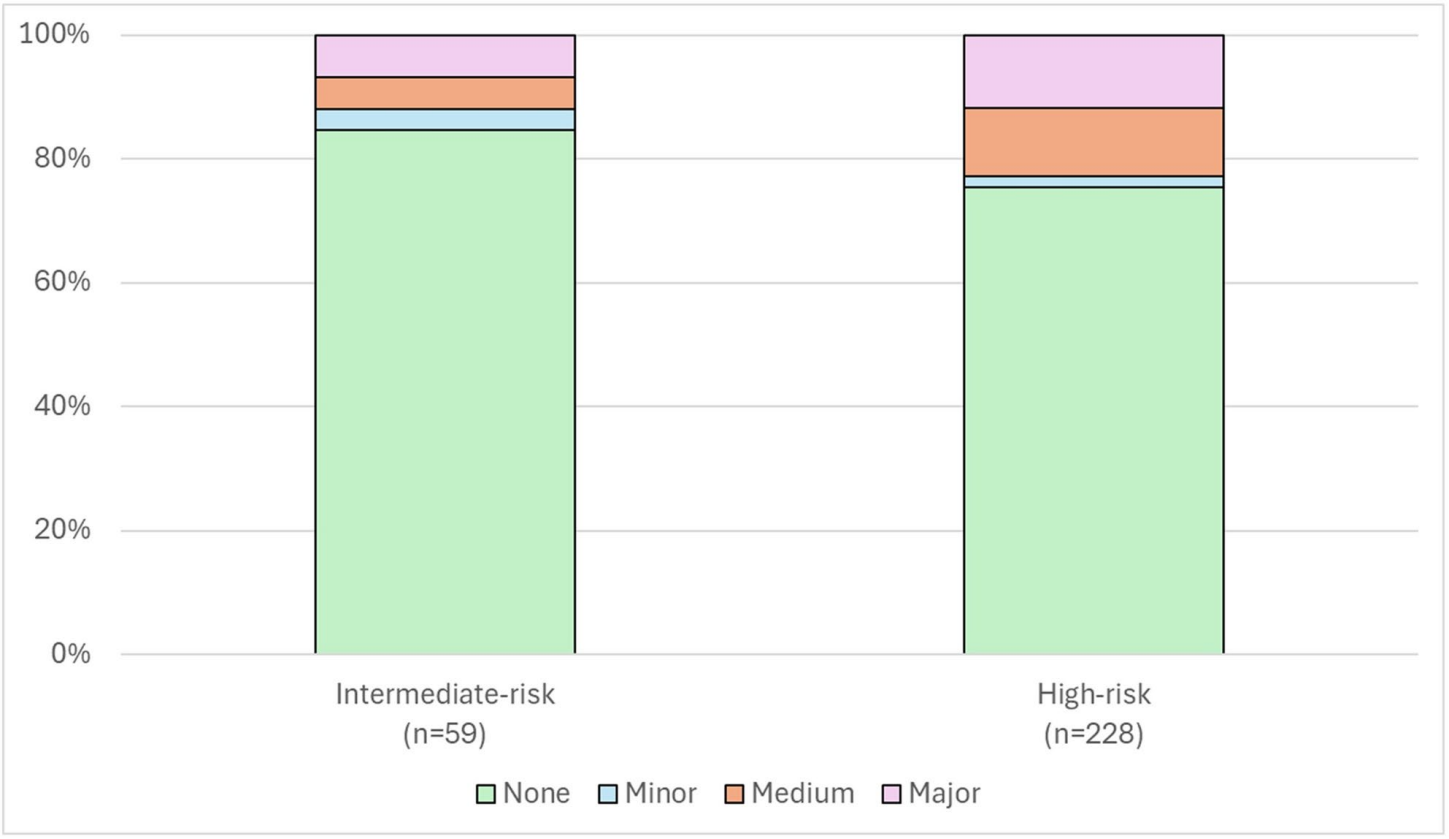
Management changes following PSMA-PET/CT stratified by risk group. Stacked bar chart showing the proportion of patients with no change, minor, medium, and major management changes in intermediate-risk and high-risk prostate cancer

In the high-risk subgroup (*n* = 228), the median PSA was 27.5 ng/mL (range 1.4–517). Negative findings across all imaging modalities were observed in 141 patients (62%), whereas positive findings were identified in 87 patients (38%). Management changes were more frequent in this group, with minor changes in 4 patients (1.8%), medium changes in 25 patients (11.0%), and major changes in 27 patients (11.8%). Additionally, imaging revealed positive findings in 31 patients (13.6%) that did not result in any changes to their clinical management. Management change stratified by risk group is shown in Fig. 3.

## Discussion

In this retrospective single-center study, we evaluated the impact of PSMA-PET/CT on clinical decision-making in the primary staging of intermediate- and high-risk prostate cancer. Our findings demonstrate that PSMA-PET/CT leads to clinically meaningful changes in management in a substantial proportion of patients, with major changes observed in 10.8% of the overall cohort. Importantly, these changes were predominantly driven by upstaging due to detection of previously unrecognized metastatic disease not identified on conventional imaging, resulting in a shift from curative to non-curative treatment intent.

The observed rate of management change is broadly consistent with previous literature. In the proPSMA trial, PSMA-PET/CT resulted in management changes in 28% of patients with high-risk disease, compared to 24.6% in our study [9]. In the phase 2/3 THUNDER trial, PSMA-PET/CT was associated with stage migration, with approximately one-third of high-risk patients being upstaged [13]. In a systematic review and meta-analysis by Jeet et al. (2023), PSMA-PET/CT resulted in management changes in 21–27% of patients in the primary staging setting [19].

Subgroup analyses further support the differential impact of PSMA-PET/CT according to disease risk. In intermediate-risk patients, management changes were relatively infrequent (overall 15.3%), which is in line with a previous study where 13.3% was reported [14]. In our study, major changes were observed in only 6.8% of intermediate-risk cases, suggesting that while PSMA-PET/CT may detect additional lesions in this group, the clinical consequences are limited in most cases. In contrast, high-risk patients demonstrated a higher rate of positive findings and management changes, including major changes in 11.8% of cases. These findings are consistent with current guideline trends and consensus recommendations, which prioritize the use of PSMA-PET/CT in high-risk disease, where accurate staging is most critical for treatment planning [18].

A key finding in our study is the substantial discordance between PSMA-PET/CT and conventional imaging. In nearly 20% of patients, PSMA-PET/CT identified lesions not detected on CT or BS, highlighting the known limitations of conventional imaging modalities [5–7]. The ability of PSMA-PET/CT to detect both small lymph node metastases and early bone involvement explains the observed upstaging and subsequent changes in treatment strategy.

Importantly, not all PSMA-positive findings translated into changes in management. In 12.2% of patients, lesions detected by PSMA-PET/CT did not alter therapeutic decisions. This highlights a critical aspect of advanced imaging: increased sensitivity does not necessarily equate to clinical utility. Treatment decisions are influenced by multiple factors, including disease extent, lesion location, patient comorbidities, and overall treatment goals. Moreover, the detection of small-volume or oligometastatic disease raises questions regarding its clinical significance and optimal management. As emphasized by Hussain et al. [15], the use of highly sensitive imaging modalities introduces the phenomenon of stage migration, where patients are reclassified without clear evidence that such reclassification improves outcomes. This underscores the need for careful interpretation of PSMA-PET/CT findings within a multidisciplinary context.

Several limitations should be acknowledged. First, the retrospective design introduces potential selection bias and limits the ability to establish causal relationships. Second, the absence of a histopathological reference standard prevents definitive assessment of the accuracy of imaging findings, particularly in cases of discordance between modalities. This is of particular relevance for [^18^F]PSMA-1007 PET/CT, which has been associated with non-specific bone uptake that may increase the risk of false-positive findings. Third, management changes were determined retrospectively based on unblinded analyses of clinical records and multidisciplinary discussions, which may not fully capture all factors influencing treatment decisions. Fourth, an additional limitation is the allowed interval of up to 90 days between conventional imaging and PSMA-PET/CT. Disease progression during this period may have contributed to discrepancies between imaging modalities in a minority of patients, particularly in patients with aggressive high-risk disease. Although an interval of up to 90 days was permitted between imaging modalities, approximately 90% of patients underwent all examinations within 30 days. Fifth, in a small proportion of patients (4.2%), anatomical assessment relied on low-dose CT acquired as part of the PET/CT examination because a separate contrast-enhanced CT was unavailable. This may have reduced the diagnostic performance of conventional imaging and should be considered when interpreting the results. Finally, the study was conducted at a single tertiary center, which may limit generalizability.

Despite these limitations, our study has several strengths, including a relatively large cohort, the inclusion of patients who underwent both conventional imaging and PSMA-PET/CT within a defined time frame, and the evaluation of real-world clinical decision-making. The consistency of our findings with existing literature supports the robustness of our results.

## Conclusion

PSMA-PET/CT has a significant impact on clinical decision-making in primary staging of prostate cancer, particularly in high-risk patients. By detecting metastatic disease not identified by conventional imaging, PSMA-PET/CT frequently leads to changes in treatment strategy, including shifts in treatment intent. However, not all additional findings translate into management changes, highlighting the importance of integrating imaging results with clinical judgment. Future prospective studies are warranted to determine whether these imaging-driven changes ultimately improve patient outcomes and to better define the role of PSMA-PET/CT in different risk groups.

## Data Availability

All data are archived according to the Swedish Act concerning the Ethical review of Research Involving Humans to attain confidentiality and are available on reasonable request.

## Abbreviations

PSMA-PET/CT: Prostate-specific membrane antigen positron emission tomography/computed tomography
BCR: Biochemical recurrence
BS: Bone scintigraphy
CT: Computed tomography
SPECT: Single-photon emission computed tomography
PSA: Prostate-specific antigen
ISUP: International Society of Urological Pathology
MDT: Multidisciplinary team
ADT: Androgen deprivation therapy
ARPI: Androgen receptor pathway inhibitor
